# Supplementary material for: Head-to-Head Comparison of Rapid and Automated Antigen Detection Tests for the Diagnosis of SARS-CoV-2 Infection
Source: J Clin Med. 2021 Jan 13;10(2):265. doi: 10.3390/jcm10020265 (PMC7828347; doi:10.3390/jcm10020265)
Supplement: Supplementary file 1 [file jcm-10-00265-s001.pdf]

Supplemental Table 1: Sensitivity and specificity of RAD and automated antigen tests across different range of RT-PCR Ct values. Results in grey provide results of all RT-PCR values including those with a Ct value > 25 (min-max range: 12.6 – 38.2).

**Sensitivity**		**No. of positive patients**							
**Ct range**	**n**	**Biotical (10 min)**	**Panbio (15 min)**	**Panbio (20 min)**	**Healgen (15 min)**	**Healgen (20 min)**	**Roche (15 min)**	**Roche (30 min)**	**Ortho**
< 15	2	2 (100%)	1 (50.0%)	1 (50.0%)	2 (100%)	2 (100%)	2 (100%)	2 (100%)	2 (100%)
> 15-20	34	33 (97.1%)	32 (94.1%)	32 (94.1%)	33 (97.1%)	33 (97.1%)	33 (97.1%)	33 (97.1%)	34 (100%)
> 20-25	22	19 (86.4%)	21 (95.5%)	21 (95.5%)	21 (95.5%)	21 (95.5%)	21 (95.5%)	21 (95.5%)	22 (100%)
**Total < 25‡**<br>**[95%CI]**	**58**	**54 (93.1%)**<br>**[83.3% to 98.1%]**	**54 (93.1%)**<br>**[83.3% to 98.1%]**	**54 (93.1%)**<br>**[83.3% to 98.1%]**	**56 (96.6%)**<br>**[88.1% to 99.6%]**	**56 (96.6%)**<br>**[88.1% to 99.6%]**	**56 (96.6%)**<br>**[88.1% to 99.6%]**	**56 (96.6%)**<br>**[88.1% to 99.6%]**	**58 (100%)**<br>**[93.8% to 100%]**
> 25	38	10 (26.3%)	11 (28.9%)	11 (28.9%)	14 (36.8%)	18 (47.4%	11 (28.9%)	11 (28.9%)	22 (57.9%)
Total (n, %)<br>[95% CI]	96	64 (66.7%)<br>[56.3% to 76.0%]	65 (67.7%)<br>[57.4% to 76.9%]	65 (67.7%)<br>[57.4% to 76.9%]	70 (72.9%)<br>[62.9% to 81.5%]	74 (77.1%)<br>[67.4% to 85.1%]	67 (69.8%)<br>[59.6% to 78.8%]	67 (69.8%)<br>[59.6% to 78.8%]	80 (83.3%)<br>[74.4%- 90.2%]
**Specificity**		**No. of negative patients**							
**Neg. RT-PCR‡**<br>**[95%CI]**	**130**	**119 (91.5%)**<br>**[85.4% to 95.7%]**	**119 (91.5%)**<br>**[85.4% to 95.7%]**	**119 (91.5%)**<br>**[85.4% to 95.7%]**	**114 (87.7%)**<br>**[80.8% to 92.8%]**	**109 (83.9%)**<br>**[76.4% to 89.7%]**	**119 (91.5%)**<br>**[85.4% to 95.7%]**	**119 (91.5%)**<br>**[85.4% to 95.7%]**	**108 (83.1%)**<br>**[75.5% to 89.1%]**
Neg. RT-PCR<br>[95%CI]	92	91 (98.9%)<br>[94.1% to 99.9%]	92 (100%)<br>[96.1%-100%]	92 (100%)<br>[96.1%-100%]	90 (97.8%)<br>[92.4%-99.7%]	89 (96.7%)<br>[90.8%-99.3%]	92 (100%)<br>[96.1%-100%]	92 (100%)<br>[96.1%-100%]	92 (100%)<br>[96.1%-100%]

Supplemental Table 2: Sensitivity and specificity of RAD and automated antigen tests across different range of RT-PCR Ct values. Results in grey provide results of all RT-PCR values including those with a Ct value > 35 (min-max range: 12.6 – 38.2).

**Sensitivity**		**No. of positive patients**							
**Ct range**	**n**	**Biotical (10 min)**	**Panbio (15 min)**	**Panbio (20 min)**	**Healgen (15 min)**	**Healgen (20 min)**	**Roche (15 min)**	**Roche (30 min)**	**Ortho**
< 15	2	2 (100%)	1 (50.0%)	1 (50.0%)	2 (100%)	2 (100%)	2 (100%)	2 (100%)	2 (100%)
> 15-20	34	33 (97.1%)	32 (94.1%)	32 (94.1%)	33 (97.1%)	33 (97.1%)	33 (97.1%)	33 (97.1%)	34 (100%)
> 20-25	22	19 (86.4%)	21 (95.5%)	21 (95.5%)	21 (95.5%)	21 (95.5%)	21 (95.5%)	21 (95.5%)	22 (100%)
> 25-30	19	6 (31.6%)	10 (52.6%)	10 (52.6%)	11 (57.9%)	13 (68.4%)	10 (52.6%)	10 (52.6%)	19 (100%)
> 30-35	6	2 (33.3%)	0 (0.0%)	0 (0.0%)	2 (33.3%)	2 (33.3%)	0 (0.00%)	0 (0.0%)	3 (50.0%)
**Total < 35‡**<br>**[95%CI]**	**83**	**62 (74.7%)**<br>**[64.0% to 83.6%]**	**64 (77.1%)**<br>**[66.6% to 85.6%]**	**64 (77.1%)**<br>**[66.6% to 85.6%]**	**69 (83.1%)**<br>**[73.3% to 90.5%]**	**71 (85.5%)**<br>**[76.1% to 92.3%]**	**66 (79.5%)**<br>**[69.2% to 87.6%]**	**66 (79.5%)**<br>**[69.2% to 87.6%]**	**80 (96.4%)**<br>**[89.8% to 99.3%]**
> 35	13	2 (15.4%)	1 (7.7%)	1 (7.7%)	1 (7.7%)	3 (23.3%)	1 (7.7%)	1 (7.7%)	0 (0.0%)
Total (n, %)<br>[95% CI]	96	64 (66.7%)<br>[56.3% to 76.0%]	65 (67.7%)<br>[57.4% to 76.9%]	65 (67.7%)<br>[57.4% to 76.9%]	70 (72.9%)<br>[62.9% to 81.5%]	74 (77.1%)<br>[67.4% to 85.1%]	67 (69.8%)<br>[59.6% to 78.8%]	67 (69.8%)<br>[59.6% to 78.8%]	80 (83.3%)<br>[74.4%- 90.2%]
**Specificity**		**No. of negative patients**							
**Neg. RT-PCR‡**<br>**[95%CI]**	**105**	**102 (97.1%)**<br>**[91.9% to 99.4%]**	**104 (99.1%)**<br>**[94.8% to 100%]**	**104 (99.1%)**<br>**[94.8% to 100%]**	**102 (97.1%)**<br>**[91.9% to 99.4%]**	**99 (94.3%)**<br>**[88.0% to 97.9%]**	**104 (99.1%)**<br>**[94.8% to 100%]**	**104 (99.1%)**<br>**[94.8% to 100%]**	**105 (100%)**<br>**[96.6% to 100%]**
Neg. RT-PCR<br>[95%CI]	92	91 (98.9%)<br>[94.1% to 99.9%]	92 (100%)<br>[96.1%-100%]	92 (100%)<br>[96.1%-100%]	90 (97.8%)<br>[92.4%-99.7%]	89 (96.7%)<br>[90.8%-99.3%]	92 (100%)<br>[96.1%-100%]	92 (100%)<br>[96.1%-100%]	92 (100%)<br>[96.1%-100%]

Supplemental Table 3: Positive predictive values (PPV), negative predictive values (NPV) and accuracies were simulated at different disease prevalence to estimate the performance of the antigenic tests in « real-life » conditions. Ct value > 33 was not considered a true positive case. *As the sensitivity and the specificity were 100% both, no confidence intervals have been calculated for the PPV and the NPV.

		Disease prevalence					
		**2%**	**5%**	**10%**	**15%**	**20%**	**25%**
**PPV**	Biotical [95% CI]	29.6% [13.8% to 52.6%]	52.0% [29.1% to 74.1%]	69.6% [46.5% to 85. 8%]	78.4% [58.0% to 90.6%]	83.7% [66.1% to 93.1%]	87.3% [72.2% to 94.8%]
	Panbio [95% CI]	63.8% [20.0% to 92.6%]	82.0% [39.2% to 97.0%]	90.6% [57.6% to 98.6%]	94.9% [68.4% to 99.1%]	95.6% [75.4% to 99.4%]	96.6% [80.3% to 99.5%]
	Healgen [95% CI]	24.6% [13.0% to 41.6%]	45.7% [27.8% to 64.8%]	64.0% [44.8% to 79.5%]	73.82% [56.3% to 86.0%]	80.0% [64.6% to 89.7%]	84.2% [70.9% to 92.1%]
	Roche [95% CI]	64.5% [20.5% to 92.8%]	82.4% [39.9% to 97.1%]	90.8% [58.4% to 98.6%]	94.02% [69.0% to 99.1%]	95.7% [76.0% to 99.4%]	96.7% [80.8% to 99.5%]
	Ortho [95% CI]	100% [N/A]*	100% [N/A]*	100% [N/A]*	100% [N/A]*	100% [N/A]*	100% [N/A]*
**NPV**	Biotical [95% CI]	99.5% [99.3% to 99.7%]	98.7% [98.1% to 99.1%]	97.3% [96.1% to 98.2%]	95.8% [93.9% to 97.2%]	94.2% [91.6% to 96.0%]	92.4% [89.1% to 94.8%]
	Panbio [95% CI]	99.6% [99.4% to 99.7%]	99.0% [98.4% to 99.3%]	97.8% [96.6% to 98.6%]	96.6% [94.8% to 97.8%]	95.2% [92.7% to 96.9%]	93.7% [90.6% to 95.8%]
	Healgen [95% CI]	99.8% [99.6% to 99.9%]	99.4% [98.9% to 99.7%]	98.7% [97.6% to 99.3%]	97.9% [96.3% to 98.9%]	97.1% [94.8% to 98.4%]	96.2% [93.1% to 97.9%]
	Roche [95% CI]	99.6% [99.4% to 99.8%]	99.1% [98.5% to 99.4%]	98.1% [96.9% to 98.8%]	97.0% [95.2% to 98.1%]	95.8% [93.4% to 97.3%]	94.4% [91.3% to 96.5%]
	Ortho [95% CI]	100% [N/A]*	100% [N/A]*	100% [N/A]*	100% [N/A]*	100% [N/A]*	100% [N/A]*
**Accuracy**	Biotical [95% CI]	95.9% [92.0% to 98.2%]	95.3% [91.2% to 97.8%]	94.2% [90.0% to 97.2%]	93.3% [88.7% to 96.4%]	92.2% [87.5% to 95.7%]	91.3% [86.3% to 94.9%]
	Panbio [95% CI]	98.7% [95.8% to 99.8%]	98.1% [95.0% to 99.5%]	97.2% [93.7% to 99.0%]	96.2% [92.4% to 98.5%]	95.3% [91.2% to 97.8%]	94.3% [90.0% to 97.2%]
	Healgen [95% CI]	94.3% [90.0% to 97.2%]	94.2% [89.8% to 97.1%]	93.9% [89.4% to 96.9%]	93.6% [89.1% to 96.6%]	93.3% [88.7% to 96.4%]	93.0% [88.4% to 96.2%]
	Roche [95% CI]	98.7% [95.9% to 99.8%]	98.3% [95.2% to 99.6%]	97.4% [94.0% to 99.2%]	96.6% [92.9% to 98.7%]	95.8% [91.8% to 98.2%]	94.9% [90.8% to 97.6%]
	Ortho [95% CI]	100% [98.1% to 100%]	100% [98.1% to 100%]	100% [98.1% to 100%]	100% [98.1% to 100%]	100% [98.1% to 100%]	100% [98.1% to 100%]
